# The Prediction of IVF Outcomes with Autologous Oocytes and the Optimal MII Oocyte/Embryo Number for Live Birth at Advanced Maternal Age

**DOI:** 10.3390/medicina59101799

**Published:** 2023-10-10

**Authors:** Jelena Havrljenko, Vesna Kopitovic, Aleksandra Trninic Pjevic, Stevan Milatovic, Tatjana Pavlica, Nebojsa Andric, Kristina Pogrmic-Majkic

**Affiliations:** 1 Ferona Fertility Clinic, Sarplaninska 19, 21000 Novi Sad, Serbia or vukosavljevicj@yahoo.com (J.H.); vesna.kopitovic@yahoo.com (V.K.); alex.trninic.pjevic@gmail.com (A.T.P.); 2 Faculty of Sciences, Department of Biology and Ecology, University of Novi Sad, Trg Dositeja Obradovica 2, 21000 Novi Sad, Serbia; nebojsa.andric@dbe.uns.ac.rs (N.A.); kristina.pogrmic@dbe.uns.ac.rs (K.P.-M.); 3 Faculty of Medicine, University of Novi Sad, Hajduk Veljkova 3, 21000 Novi Sad, Serbia; milatstevan@gmail.com

**Keywords:** infertility, in vitro fertilization, advanced age, delayed childbearing, oocyte, embryo

## Abstract

*Background and Objectives:* Delayed childbearing in advanced age might be associated with a low prognosis for achieving pregnancy. Therefore, it is important to establish a predictive tool that will optimize the likelihood of a live birth at advanced age. *Material and Methods:* The retrospective study was conducted at the Ferona Fertility Clinic in Novi Sad (Republic of Serbia), between January 2020 and May 2021. The survey included 491 women aged ≥35 who met the inclusion criteria and who were subjected to an IVF (in vitro fertilization) treatment cycle. *Results:* The average number of retrieved oocytes, MII (metaphase II) oocytes, and developed embryos significantly decreased in advanced age. Age was also found to have a significant adverse effect on pregnancy and live birth rates. In women aged ≥35, 10/12 MII oocytes or 10/11 embryos are required for reaching an optimal live birth rate/cumulative live birth rate. Optimal CLBR (cumulative live birth rate) per one oocyte was achieved when 9 MII oocyte were retrieved. *Conclusions:* The study indicates that the cut-off for increased risk is ≥42 year. However, despite low live birth rates, autologous IVF for these women is not futile. An increase in the number of retrieved mature oocytes and a generation of surplus cryopreserved embryos could reinforce LBR (live birth rate) and CLBR. Clinicians should be very cautious in counseling, as autologous IVF may only be applicable to women with good ovarian reserve.

## 1. Introduction

In the last few decades, developed countries have recorded an increasing trend in delayed childbearing [1]. The reasons can be associated with the rigorous pursuit of women’s educational and career goals, highly effective contraceptive strategies, and the misleading idea that in vitro fertilization (IVF) can compensate for the natural decline in infertility associated with aging [2]. This trend has also been observed in Serbia with the main reasons for this phenomenon being existential problems; dissatisfaction with the political context and an uncertain future; difficulties in achieving a stable relationship; women bearing the burden of heavy family duties; and the mentalities of individualism, hedonism, and consumerism [3]. In Serbia, the average age of a mother delivering her first child has risen from 26.7 years of age in 2001 to 30 in 2018 [4]. As data from the Statistical Office of the Republic of Serbia show, the total fertility rate was 1.52 or 28% below the replacement level in 2019, as well as below the European average of 1.6 children per woman [5].

The postponement of childbearing to advanced age can result in reduced ovarian reserve, which is the main factor of female infertility in Serbia [6]. When applicable, assisted reproduction and in vitro fertilization (IVF) are the standard approach of coping with female infertility [7]. In Serbia, the first IVF baby was born in 1987 [6], while the state has financially supported these procedures since 2006 [8]. At present, both private and public health institutions in possession of a permit from the Directorate for Biomedicine of the Ministry of Health of the Republic of Serbia can perform IVF [9], while the Republic Fund for Health Insurance covers the costs of unlimited IVF trials for women up to 45 years of age [10]. According to the latest European monitoring, the majority of women in Serbia (39.6%) undergoing fertility treatment were aged ≥40; 31.3% of them were between 35 and 39, while 29.2% were women aged <34. Data from this monitoring showed that in Europe, the average percentage of women aged ≥40 undergoing IVF was 20.9%, the average percentage for the age group 35–39 was 31.3%, and 29.2% of treated women were in the youngest group—i.e., <34 years of age [11].

In assisted reproduction, maternal age is among the strongest predictors of success [12]. Advanced maternal age (AMA) is defined as the turning point at which pregnancy rates significantly decline [13], but there is no universal consensus on the exact maternal age at which the risk increase for adverse pregnancy outcomes becomes clinically significant [14]. This polemic is partly due to the fact that the effects of increasing age occur as a continuum rather than a threshold effect, and declining fertility is an individual event that differs for every woman [15]. The majority of studies have reported outcomes in women aged ≥35 years [16,17,18] or women aged ≥40 years [19,20,21] as the cut-off for increased risk.

Despite the continuous progress of assisted reproductive technologies (ART), infertility is still a problem for women of AMA. Live birth rates after IVF remain low, especially in women over 40 [22]. In the United States of America, only 3.5% of births occur in women aged 40 years or older [23]. A possible explanation for this fertility reduction can be found in declining ovarian reserve combined with age-related decreased endometrial receptivity and increasing aneuploidy rates in oocytes [24]. Ubaldi [2] found that women older than 35 experience a dramatic increase in the embryo aneuploidy rate, from a 30% baseline production up to 90% in their late 40s prior to menopause. Furthermore, the chance of producing a chromosomally normal blastocyst might be even lower than 5% in women older than 43. In support of this claim, the ART calculator, which is a clinical predictive model used to estimate the number of mature oocytes needed to obtain at least one euploid embryo, indicates that 13, 16, and 19 oocytes are needed for women aged 38, 39, and 40. In contrast, only 5 and 6 oocytes are required for patients aged 33 and 34 [25]. Numerous other studies have investigated the relationship between the number of oocytes retrieved during ovarian stimulation and live birth outcomes. Evidence suggests that retrieving a higher number of oocytes theoretically improves the chances of achieving a live birth by increasing the number of embryos available for transfer [26]. Furthermore, improvements in cryopreservation technology have dramatically increased the efficiency of frozen embryo transfers, hence improving the overall probability of obtaining a live birth after multiple transfers of embryos originating from a single cycle of ovarian stimulation (cumulative live birth rates) [27,28]. Over the last decades, excellent pregnancy and live birth rates have made the use of oocyte donation an indispensable part of assisted reproduction for women with ovarian reserve depletion [29]. Thus, while it is possible to overcome the biological clock for AMA women, the decision to renounce genetic maternity is crushing and generally one of the most momentous moments in life. Contrary to high live birth rates, donation should be considered a treatment failure, since it represents a treatment of second choice.

Noticeably, research that could lead to IVF outcome improvements in older women using autologous oocytes has been abandoned. Estimates show that the proportion of women over 40 years of age undergoing IVF worldwide with their own oocytes is at least 25% [30]. Most evidence of the clinical outcomes for advanced age women has been derived from outdated analyses. Limited studies concerning autologous IVF in women aged ≥40 refer to live birth rates after three or more repeated cycles [31]; however, this data may not represent everyday clinical practice. Outcomes after a single stimulation cycle could be more meaningful for patient counseling.

Developing a counseling tool that predicts the likelihood of achieving at least one live birth in women of an advanced age with their own oocytes is pivotal. Thus, this study aimed to evaluate clinical outcomes in women aged ≥35, so as to investigate if women of very advanced age (≥45) have a reasonable chance for childbearing with their own oocytes and to calculate the required number of mature oocytes and embryos for achieving optimal live birth rates in women aged ≥35.

## 2. Materials and Methods

This retrospective study was carried out in a private clinic between January 2020 and May 2021. The clinic’s medical records were obtained from patients subjected to an IVF treatment cycle. The investigation was performed with the approval of the Ethics Board of the Ferona Fertility Clinic (No 1125-1/1-19) in accordance with the Helsinki declaration.

**Figure 1:**
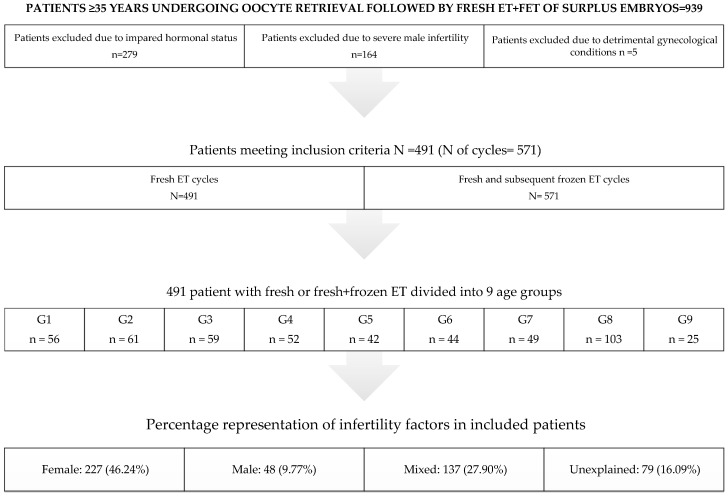
Demographic characteristics of the study group selection process.

The study included women aged ≥35 years who underwent follicle aspiration followed by the fertilization of autologous oocytes and a fresh embryo transfer (ET), as well as the subsequent frozen embryo transfer (FET) of surplus vitrified embryos. In accordance with ESHRE (European Society for Human Reproduction and Embryology) recommendations, all patients referred to oocyte retrieval tested negative for COVID-19 [32]. Patients designated for the freeze-all strategy, donation, or preimplantation genetic testing (PGT) were not considered in this analysis, nor were patients with no mature (metaphase II) oocytes after retrieval or embryos for ET. Other exclusion criteria were impaired hormonal status, particularly anti-Müllerian hormone-AMH and follicle stimulating hormone-FSH (only patients with FSH < 15 mlU/mL and AMH > 0.5 ng/mL and <4.0 ng/mL were analyzed). Also excluded from the research sample was severe male infertility according to the WHO’s [33] categorization (azoospermia, cryptozoospermia, surgically retrieved spermatozoa, necrozoospermia, <4% morphologically normal sperm cell according to Krüger criteria), as well as detrimental gynecological conditions such as polycystic ovary syndrome (PCOS), hydrosalpinx, severe endometriosis, and untreated uterine issues (septum, myomas, and polyps). The demographic characteristics of the study group selection process are given in Figure 1.

The treatment protocol for eligible patients included ovarian stimulation according to the flexible GnRH antagonist protocol followed by intracytoplasmic sperm injection (ICSI). Ovulation was triggered with recombinant hCG when at least one dominant follicle ≥ 18 mm in diameter was observed and the estradiol (E2) level had reached an appropriate level. Oocyte pick-up was performed 36 *h* after the ovulation trigger, and ICSI was applied to metaphase II oocytes. During the period in which the patients underwent IVF, national reimbursements covered the financial costs of 3 IVF cycles and a maximum of 6 frozen embryos [34]. Due to these limitations, most of the patients opted for cryopreservation at the blastocyst stage, so that the complete embryo transfer was performed on day 3 after oocyte retrieval, while surplus embryos were cultivated until day 5/6 and frozen at the blastocyst stage. The Law on Biomedically Assisted Reproduction in Serbia [35] regulates embryo transfer to a maximum of 3 embryos. After counseling with a clinician, the patient made the final decision on the number of embryos. Embryo quality was assessed in relation to blastomere number and regularity, fragmentation and multinucleation contribution, blastocyst expansion, morphology of inner cell mass (ICM), and trophoectoderm (TE). According to our scoring system, an A quality embryo grade refers to 6–8 cell embryos on day 3 with equal and regular blastomeres, no multinucleation, and up to 10% fragmentation. After ultrasound guided ET, vaginal/oral progesterone was administrated for luteal support. FET cycles were performed in artificial hormone replacement cycles and after an ultrasound-guided transfer of a maximum of 2 warmed blastocysts, progesterone luteal support was applied. Successful implantation was established with an increased serum-βhCG level, while the fetal heartbeat was confirmed with a vaginal ultrasound performed at the 6th gestational week.

The patients were categorized into nine age groups: G1 age 35, G2 age 36, G3 age 37, G4 age 38, G5 age 39, G6 age 40, G7 41, G8 age 42–44, and G9 age ≥ 45. Each group was categorized according to the decimal age. For example, the group of patients aged 35 years included all patients between 35.0 and 35.9.

The following main IVF outcomes for these age groups were reviewed and included: the number of retrieved oocytes, the number of mature (MII) oocytes, fertilization rates, the number of cleaved embryos, the rate of A quality embryos, the number of transferred embryos, cumulative positive βhCG rates, clinical pregnancy rates, and live birth and miscarriage rates. Further, additional analysis was done in order to calculate an optimal number of MII oocytes and created embryos for reaching reasonable live birth, cumulative live birth rates, and the number of required MII oocytes for cumulative live birth per one oocyte.

Statistical analysis was done using SSPS software version 25.0. Differences among age groups were tested using ANOVA, a chi-squared test, and post hoc contrast analysis. Frequency and binomial analyses were applied for assessing deviations from theoretically expected distribution. Polynomial regression was used for prediction analysis. The comparison was based on the latest ESHRE reported clinical pregnancy rate of 32.1% per ET after ICSI and the delivery rate of 23.9% per ET after ICSI in women ≥ 35 [11].

## 3. Results

An increase in women’s age resulted in a decrease in the average number of retrieved oocytes [F(8.481) = 5.11, *p* < 0.001], the average number of MII oocytes [F(8.481) = 2.92, *p* < 0.01], and the average number of developed embryos across the various age groups (Table 1). Contrast post hoc testing determined a general downward trend between the following age groups: G1 vs. G8, G1 vs. G9, G2 vs. G8, G2 vs. G9, G3 vs. G8, G7 vs. G8, and G7 vs. G9.

Fertilization rates and the percentage of quality embryos remained stable across all age groups, and statistical differences were not detected. The mean number of transferred embryos among different groups also had no significant effect.

Age was found to have a significantly decreasing effect on positive βhCG rates (χ^2^ = 20.62, *p* < 0.001), clinical pregnancy rates (χ^2^ = 35.14, *p* < 0.001), and live birth rates (χ^2^ = 31.84, *p* < 0.001). With increasing age, positive βhCG rates dropped from 49.10% (G1) to 24% (G9), leading to a significant decrease in clinical pregnancy rates from 38.2%(G1) to 16% (G9), and to a significant decrease in live birth rates from 38.2% (G1) to 8% (G9). On the other hand, a significant increase in miscarriage rates was observed with aging (χ^2^ = 19.54, *p* < 0.01). A similar fading trend among different age groups was observed with clinical pregnancy and live birth rates after the subsequent FET of surplus embryos.

Frequency analysis and a binominal test were applied to investigate if there was a significant decrease in positive βhCG, clinical pregnancy rates, and live birth rates and an increase in miscarriage rates in a particular age group after fresh ETs (Table 2). The analysis indicated a significant decline of positive βhCG in older age groups (G5-G9), whereas this downfall trend was not observed in younger age groups. A major decrease was detected in G8, followed by G7, G5, G9, and G6. In terms of clinical pregnancy and live birth rates, a statistically significant difference was evident among all age groups except the youngest group, G1. The most evident decrease was observed in group 8, followed by G7, G6, G5, G9, G4, G2, and G3. A significant increase in miscarriage incidences was found in all groups except G1, G4, and G6. Group 8 showed the most apparent increase and groups G3, G2, G5, G7, and G9 followed a downward trend.

**Figure 2:**
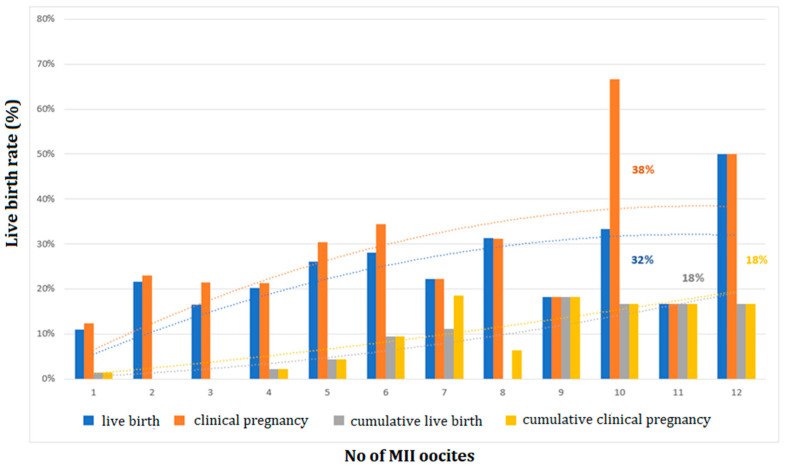
The clinical pregnancy rate after fresh ET cycles (*n* = 491) and cumulative clinical pregnancy rate after subsequent FET cycles (*n* = 571); the live birth rate after fresh ET cycles (*n* = 491) and cumulative live birth rate after subsequent FET cycles (*n* = 571), according to the number of retrieved MII oocytes.

**Figure 3:**
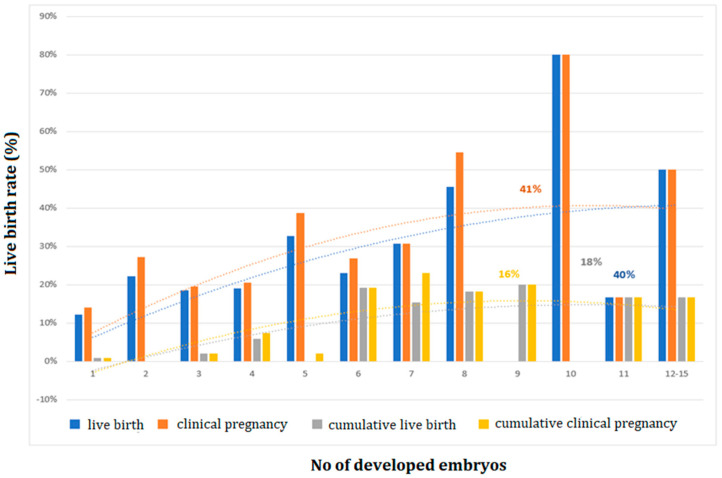
The clinical pregnancy rate after fresh ET cycles (*n* = 491) and cumulative clinical pregnancy rate after subsequent FET cycles (*n* = 571); the live birth rate after fresh ET cycles (*n* = 491) and cumulative live birth rate after subsequent FET cycles (*n* = 571), according to the number of created embryos.

Polynomial analyses were applied across all age groups to investigate the optimum levels of LBR and CLBR in association with the number of MII oocytes and created embryos as well as LBR per one oocyte. Clinical pregnancy rate (CPR) per stimulated cycle reached an optimum level (38%) when 10 MII oocytes were retrieved, and LBR reached an optimum level (32%) in the case of 10 MII oocytes, after which it plateaued (Figure 2). In addition, the CPR and CLBR of fresh and subsequent FETs optimized (18%) at 12 MII oocytes, then evened out (Figure 2). An analysis of the embryo number demonstrated that CPR per stimulated cycle reached an optimum level (41%) when 10 embryos were created, or 9 embryos (16%) after a subsequent FET of surplus embryos (Figure 3). LBR was optimal (40%), with 11 embryos in fresh stimulated cycles or with 10 embryos after subsequent FET (18%) (Figure 3).

**Figure 4:**
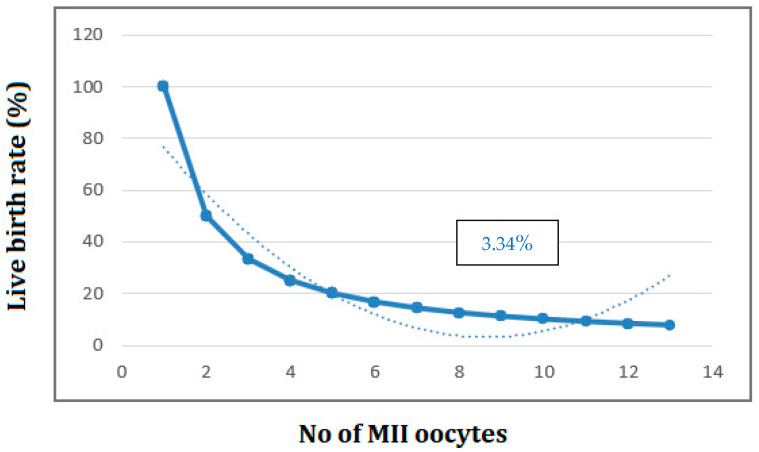
Cumulative live birth rates per oocyte according to retrieved MII oocytes. Number of cycles = 571.

The CLBR after fresh ET and subsequent FET per oocyte declined down to 3.34% when 9 MII oocytes were collected (Figure 4).

## 4. Discussion

This study shows that AMA is the main factor associated with rapid fertility decline and chance of childbearing, as is confirmed in other recent studies [22]. Our analysis is in complete agreement with other studies [36,37,38], suggesting a decrease in the number of retrieved oocytes as well as the number of MII oocytes with women’s aging. Although the results revealed no differences in fertilization potential among the age groups, there was a direct correlation between the women’s age and the clinical outcome: the higher positive βhCG rates, the higher clinical pregnancy rates and live birth rates in younger patients and the higher miscarriage rates in older patients. In comparison with the latest ESHRE Registry (2018) which reports the average pregnancy rate of 32.1% per ET after ICSI and a delivery rate of 23.9% per ET after ICSI in women ≥ 35 years of age [11], the data obtained in our study indicate that the greatest decline in clinical outcomes occurs in women in the ≥42 age group. The study also shows that women aged ≥42 are at higher risk for miscarriage and pregnancy complications when compared with younger women, which is consistent with other reports [39,40]. According to the reports, the mode of delivery was also significantly influenced by maternal age. With increasing age, the incidence of caesarean section significantly increased, and the highest percentage (61%) was found among women over 40 years of age [41]. These findings reinforce the role of the age factor as crucial for ART outcomes and confirm other research findings that women over 40 years of age are likely to face more difficulties in achieving pregnancy [15,42,43].

Many studies emphasize that not only does the number of retrieved oocytes affect IVF outcomes, but also the quality of oocyte in women of advanced age [44,45]. Factors contributing to compromised oocyte quality in older women include mitochondrial dysfunction and abnormal gene expression. The loss of mitochondrial activity in oocytes obtained from AMA women undergoing IVF could lead to slower cell divisions that affect embryonic development and impair pregnancy rates [46]. Embryos produced from these oocytes are usually of poor quality and exhibit lower implantation potential [47]. This study found no significant difference in embryo quality between older and younger patients, but a statistical difference in the number of developed embryos was detected between younger and older age groups. Aneuploidy is known to occur in both good and poor quality embryos [48,49]. Therefore, we may assume that although there were no differences in embryo quality in our study, lower clinical outcomes in older patient groups could be due to increased aneuploidy.

As the embryo aneuploidy rate increases with age, one of the main challenges for women of advanced age is obtaining enough available embryos for better embryo selection. Considering that more than 90% of age-related embryo aneuploidy are of maternal origin and caused by chromosomal misaggregation during oogenesis [12], many scientists strongly propose preimplantation genetic testing for aneuploidies (PGT-a) as a valid approach for increasing live birth rates in advanced age women [50]. Recent publications revealed that PGT-a in women aged ≥44 may lead to a delivery rate of 8% per cycle [51]. Thus, an important assignment in treating advanced age women is obtaining more oocytes in order to have a chance of finding euploid embryos [13]. However, for women of advanced age it is often impossible to produce many embryos due to low ovarian reserve [52].

While concern for the optimization of oocyte or embryo yield rises, it is still unknown how many oocytes will optimize live birth rates and cumulative live birth rates at present [53]. This study showed similar results with other studies demonstrating that a larger number of oocytes and a generation of surplus cryopreserved embryos are associated with higher LBR and CLBR [26,54]. Contrary to previous published analysis which investigated the overall number of retrieved oocytes, our study enhanced the number of MII oocytes, given that the vast majority of retrieved oocytes were either immature or incapable of creating a good embryo [27]. In answer to a highly clinically relevant question, this study showed that the required number of MII oocytes and embryos for reaching an optimal LBR/CLBRis 10 MII/12 MII oocytes or 11/10 embryos in women aged ≥35. This outcome, when jointed with the disclosure that the highest CLBR per one oocyte was achieved when at least 9 MII oocytes were fertilized, is a potential prognostic tool for predicting IVF outcomes in women of advanced age.

Although the number of MII oocytes and the number of created embryos in this study did not reach the calculated optimum in any of the age groups, live birth and cumulative live birth rates were in the range of the worldwide average. Thus, we may assume that maximizing the number of oocytes retrieved from a single ovarian stimulation and a generation of a large number of embryos would be beneficial in achieving fertility goals in advanced age. An individualization of the IVF treatment and tailored stimulation protocols based on a predicted ovarian response could be a vital step towards a successful outcome. The accumulation of embryos or oocytes from several stimulation cycles has also been suggested to enhance the possibility of childbirth in women with a limited ovarian reserve [55,56]. Referring to recent articles, there is a lack of evidence that the transfer of multiple embryos obtained from several cycles increases pregnancy rates in AMA patients [57]. Previous studies have highlighted that extended embryo culture until the blastocyst stage is the best way to select embryos with the highest implantation potential in patients with good ovarian reserve [58]. Moreover, frozen blastocysts provide similar or even better implantation rates compared to fresh embryo transfers [59]. However, some patients, especially AMA women, may have no blastocysts for transfer or cryopreservation regardless of a proper ovarian reserve, considering that not all cleavage stage embryos will be able to reach the blastocyst stage due to increased aneuploidy rates [43]. Only 80 out of 491 patients from this study had a cryopreserved blastocyst after a fresh embryo transfer of cleavage embryos, while 21 out of 80 patients who did not achieve a pregnancy after fresh embryo transfer had a clinical pregnancy (25.25%) and 19 had childbirth (23.75%) after a subsequent FET of a frozen blastocyst. These outcomes correlate with other results [60] suggesting that the blastocyst embryo culture is not harmful in AMA patients with good ovarian reserve, and this could be a useful and effective strategy when combined with reaching an optimal oocyte number.

In contrast to recent publications on IVF outcomes in women of very advanced age [61,62], this study points to a higher clinical pregnancy rate and live birth rate in women ≥45 years of age. However, it should be highlighted that this age group only included a small number of patients. We suppose that the inclusion of women with unexpectedly good ovarian reserve for their age and their potential to produce enough good quality embryos might have resulted in childbirth at a very advanced age with autologous oocytes.

Despite the poor clinical prognosis, some patients still attempt to explore all treatment options with their own oocytes before committing to oocyte donation [63]. Regardless of decreased oocyte yield and low live birth rates, our study demonstrates that autologous IVF for women aged ≥42 is not futile. According to the ASRM (American Society for Reproductive Medicine) Ethics Committee, futility in infertility is a live birth chance of less than 1% [64]. Nevertheless, clinicians should be very careful in counseling, as it may only be applicable to women with good ovarian reserve. Patients should be clearly informed about their clinical prognosis.

As female age cannot be modified, if expective oocyte and embryo yield is hard to reach, oocyte donation should be encouraged. According to Cimadomo [12], 35 should be the lowest age threshold to define AMA, and 45 should be considered the highest age threshold to undergo IVF with one’s own oocytes. Autologous IVF should not be repeated if a patient has experienced a recurrent failure of follicle growth, fertilization, or embryonic development [65].

## 5. Conclusions

The results overall indicate that AMA is associated with risks for IVF outcomes. The risks are strongest with the number of retrieved oocytes, the number of MII oocytes, the average number of developed embryos, βhCG rates, clinical pregnancy rates, live birth rates, and higher miscarriage rates. The greatest decline in clinical outcomes appears in the group of women aged ≥42. Obtaining an optimal number of retrieved MII oocytes and created embryos could increase the chance for a live birth. These findings should be considered when counseling women of advanced maternal age. Recommendations and the treatment of these patients should take into consideration their age and the expected ovarian response. Further improvements to treatments, as well as reinforcing research, are needed when treating advanced aged women who reject oocyte donation as a solution.

## Figures and Tables

**Table 1:** IVF outcomes in various age groups.

AG	No of Retrieved Oocytes Mean ± SD	No of MII Oocytes Mean ± SD	FR %±SD	No of Cleaved Embryos Mean ± SD	A Quality Embryo Rate %±SD	No of Transferred Embryos Mean ± SD	Positive βhCG (%)	CPR/ CCPR (%)	LBR/ CLBR (%)	MR (%)
35	7.18 ± 3.86	4.78 ± 2.35	86.29 ± 17.66	3.89 ± 2.03	62.35 ± 36.51	2.02 ± 0.62	49.10	38.20/45.5	38.20/43.6	0.0
36	6.84 ± 3.81	4.58 ± 2.89	85.65 ± 18.60	3.63 ± 2.31	56.31 ± 36.14	2.02 ± 0.76	46.77	33.33/37.1	33.33/35.5	1.6
37	6.66 ± 3.55	4.38 ± 2.33	83.94 ± 20.17	3.38 ± 1.78	58.41 ± 37.63	2.02 ± 0.76	43.10	34.50/39.7	32.80/37.9	1.7
38	6.10 ± 3.17	4.13 ± 2.00	82.32 ± 21.03	3.21 ± 1.81	65.71 ± 37.52	2.04 ± 0.71	40.40	28.80/28.8	28.80/28.8	0.0
39	5.45 ± 3.07	3.60 ± 2.39	84.89 ± 19.99	2.71 ± 1.90	65.14 ± 39.63	2.02 ± 0.81	28.60	19.00/21.4	16.70/19.0	2.4
40	5.82 ± 3.82	4.20 ± 2.91	85.99 ± 19.30	3.27 ± 2.50	58.49 ± 38.15	1.91 ± 0.71	31.80/	15.90/20.5	15.90/20.5	0.0
41	6.98 ± 4.21	4.45 ± 3.12	85.16 ± 19.49	3.73 ± 2.93	56.41 ± 39.34	2.02 ± 0.77	28.60	16.30/20.4	10.20/14.3	6.1
42–44	4.58 ± 2.92	3.25 ± 2.05	87.63 ± 17.82	2.62 ± 1.58	56.59 ± 40.49	2.11 ± 0.93	16.50	13.60/15.5	7.80/9.7	5.8
≥45	4.20 ± 2.61	3.52 ± 2.37	90.22 ± 17.20	2.88 ± 1.81	56.43 ± 35.02	2.04 ± 0.94	24.00	16.00/15.4	8.00/7.7	8.0
*p*	<0.001	<0.01	NS	<0.01	NS	NS	<0.001	<0.001	<0.001	<0.01

Abbreviations: AG—age group; FR—fertilization rate; CPR—clinical pregnancy rate; LBR—live birth rate; MR—miscarriage rate; CCPR—cumulative clinical pregnancy rate; CLBR—cumulative live birth rate; NS—not significant.

**Table 2:** Binomial analysis of clinical outcomes across various age groups.

Age Group	Positive βhCG			Clinical Pregnancy			Live Birth			Miscarriage		
	Yes	χ^2^	*p*	Yes	χ^2^	*p*	Yes	χ^2^	*p*	Yes	χ^2^	*p*
G1(35y)	49.09%	0.02	0.89	38.18%	3.07	0.08	38.18%	3.07	0.08	0%	-	1
G2(36y)	46.77%	0.26	0.61	33.33%	7.81	<0.01	33.33%	7.81	<0.001	1.61%	58.01	<0.001
G3(37y)	43.10%	1.1	0.29	34.48%	5.59	0.02	32.73%	6.9	<0.01	1.72%	54.07	<0.001
G4(38y)	40.38%	1.92	0.16	28.85%	9.31	<0.01	28.85%	9.31	<0.01	0%	-	1
G5(39y)	28.57%	7.71	<0.01	19.04%	16.1	<0.001	16.67%	18.67	<0.001	2.38%	38.1	<0.001
G6(40y)	31.81%	5.82	<0.05	15.91%	20.46	<0.001	15.91%	20.46	<0.001	0%	-	1
G7(41y)	28.57%	9	<0.01	16.33%	22.22	<0.001	10.20%	31.04	<0.001	6.12%	37.75	<0.001
G8(42–44y)	16.50%	46.22 ^a^	<0.001	13.59%	54.61 ^a^	<0.001	7.77%	73.49 ^a^	<0.001	5.83%	80.4 ^a^	<0.001
G9(≥45y)	24.00%	6.76	<0.01	16.00%	11.56	<0.001	8.00%	17.64	<0.001	8.00%	17.64	<0.001

Abbreviations: ^a^—the most significant difference compared to expected value.

## Data Availability

All data involved in this work will be made available by the corresponding author upon request (tatjana.pavlica@dbe.uns.ac.rs).
